# From inputs to impact: Study protocol for developing a framework for laddered graduate certificate programs

**DOI:** 10.1371/journal.pone.0345557

**Published:** 2026-04-02

**Authors:** Lorelli Nowell, Kate Beamer, Sara Dolan, Lisa Fedoruk, Anne-Marie McLaughlin, Kimberley Grant, Alix Westgard

**Affiliations:** 1 Faculty of Nursing, University of Calgary, Calgary, Alberta, Canada; 2 Faculty of Health Disciplines, Athabasca University, Athabasca, Alberta, Canada; 3 Department of Community Health Sciences, Cumming School of Medicine, University of Calgary, Calgary, Alberta, Canada; 4 Faculty of Social Work, University of Calgary, Calgary, Alberta, Canada; 5 Taylor Institute for Teaching and Learning, University of Calgary, Calgary, Alberta, Canada; PLOS: Public Library of Science, UNITED KINGDOM OF GREAT BRITAIN AND NORTHERN IRELAND

## Abstract

Present-day postsecondary institutions are increasingly expected to implement innovative solutions to support the learning and development of professionals while providing credentials responsive to both academic and non-academic careers. Laddered graduate certificate programs, sometimes called stackable certificate programs or micro-credential graduate programs, have been increasingly implemented across higher education settings. These programs are designed to upskill the workforce by providing a professional, flexible learning experience with the goal of a mutually beneficial relationship between industry and academia. Although substantial emphasis has been placed on developing practical, flexible, and adaptable certificate programs to meet the professional and personal needs of students, there is a distinct lack of evidence-based guidance within existing literature to inform quality program development and ongoing innovation within these types of programs. The purpose of this paper is to describe the methods for a comparative case study to better understand the experiences and perceptions of students, educators, alumni, program leaders, and industry partners participating in graduate certificate programs. Online surveys, interviews, and focus groups will be conducted with these valuable partners to gain better insight into teaching and learning experiences, program structures, desired outcomes, areas for improvement, and pedagogical innovation. Data sources, including artifacts, documentation, and archival records regarding accreditation processes, industry partners, curriculum development and implementation, and student work, will be used to provide valuable context. Quantitative and qualitative data will be integrated and used to develop an evidence-informed framework that articulates the conditions that optimise student learning and program innovation within laddered graduate certificate programs.

## Introduction

Laddering, in terms of education, refers to earning two or more credentials in the same or similar academic field over a period of time [1]. These certificate programs are often referred to as “laddered,” “stacked,” or “latticed” programs due to the sequential ability to accumulate multiple certifications building towards a degree [1]. Laddered, or stacked, credentials emerged as educational options in the early 21^st^ century and are becoming increasingly popular as an accessible educational option [2]. Since their introduction, laddered certifications have not only increased in availability but are also expanding into additional fields and disciplines [3]. Laddering is available at different levels of postsecondary education, including at colleges and universities and can result in certifications such as associate’s, bachelor’s, and master’s degrees [4]. Depending on program type, laddering can result in varying forms of certification, including academic degrees, educational or professional certificates, and occupational licenses [4]. While certification laddering can take on many forms, the most common is a progressive pathway, where “each credential signifies another step, or rung, along a career ladder” [5 p72]. Industry changes over the past decades have contributed to increased demand for workforce upskilling [6,7]. Employers have reported challenges filling positions due to the lack of specific skills required [5]. This is especially true with the advancement of technology, often requiring additional training to be technologically current [8]. These gaps in the labour market represent a major contributing factor in the expansion of laddered certificate programs [9].

The benefits of laddered programs are numerous. A review of existing literature indicated that laddered programs could have economic benefits to students, noting both the ability to maintain employment while being in school and often receiving a wage increase post-program [1]. Laddering, or stacking credentials, can allow students to complete a degree in increments or pursue certifications, while still being a part of the workforce [5]. In relation, laddered certification provides an accessible, short-term, lower-cost path to attaining an education [1]. These programs are ideal for mature and working students, as they can increase their education without the long-term time commitment required by degrees [9]. Increased employment opportunities and employment stability were also noted as a benefit [1]. As laddered certificates often take less time to complete, they have higher graduation rates in comparison to degrees [9]. Financial benefits were also noted, as tuition costs are often lower and staggered among certificates [10], which allows for increased diversity among students enrolled in such programs. While pursuing a credential, it was noted that students enrolled in a laddered certificate program often incur fewer fees and less educational debt than those pursuing traditional degrees [1].

Certificate programs have long been utilized in community colleges as stepping stones for students without higher education degrees to help increase industry-relevant knowledge, enhance skills, and foster greater employability [1,11–13]. The same principles are more recently being applied to graduate programs as educational institutions are recognizing the need to support non-traditional, mature learners seeking to update their knowledge and skills to support career advancement [14,15]. Certificate programs have been cited as a solution to address rapidly changing student demographics, consumerism, and evolving technology [6,16], allowing students to earn certificates in topic areas that best align with needs in their chosen industry, customize their learning to their desired career trajectory [6] and even ladder to graduate degrees [15]. These programs are often developed to be relevant to the workplace [17], consumed in smaller timeframes, and used to target evolving industry needs [7]. Professionals in the workforce can increase their perceived employability and human capital by upskilling with certificates that align with their career interests [18,19]. Furthermore, laddered certificates and micro-credentials may be a more approachable solution for continued education to gain valuable knowledge and skills that will be seen as an asset in their industry [20].

Although laddered graduate certificate programs are increasingly present, it is essential to understand what factors promote quality and innovation within these programs. This is especially essential, considering the lack of literature centred on laddered certificate programs at a graduate level. Increased data on program implementation, policies, funding and faculty buy-in, and student outcomes could create a level of consistency and best practices among laddered certificate programs [4]. Although prior research has reported the benefits of laddering programs, these empirical studies have focused on student experiences within community college settings [2,21]. These studies have found that laddering certificates are associated with increased employment opportunities [21], and greater flexibility in program design [2], which may enable students to maintain their professional designations while advancing their education [22]. Evidence from community college contexts has also indicated that student participation in laddered credentials is associated with higher graduation rates and completion of multiple credentials [10,23]. Laddered graduate certificate programs aim to provide an output value that offsets the students’ time and money necessary to obtain the certificate or degree [9] and it is essential these programs are structured in a way to best meet the needs of all partners [24], and have a nimbleness to foster innovative curriculum that responds to industry changes efficiently and effectively.

### Specific purposes

A mixed methods comparative case study analysis will be completed to:

1. Describe the structural inputs, processes, and proximal outputs of laddered graduate programs.
2. Compare the structural inputs, processes, and proximal outputs across laddered graduate programs over time.
3. Describe and examine the impact of structural inputs and processes on the laddered graduate program proximal outputs.
4. Create a framework to support quality and innovation factors that can be integrated into new and existing laddered graduate certificate programs.

## Methods

The details and complexity of the questions being studied necessitate using a combination of quantitative and qualitative data collection/analysis methods [25]. A comparative case study mixed-methods approach will be employed for data collection, analysis, and integration of quantitative and qualitative information to examine how laddered graduate programs are structured (inputs), implemented and experienced (process), and what significant outputs can be expected. Participant recruitment and data collection will begin in November 2025 and run until March 2030, with final results expected in August 2030.

This research will be approached from a pragmatic philosophical and epistemological standpoint, underpinned by an integrated Knowledge Translation (iKT) theoretical lens (Nowell, 2015), and an Input Process Output (IPO) model [26]. Through varied lenses each laddered graduate certificate program (case) will be evaluated. Multiple data sources will be used to develop and interpret a description of each case and compare cases for convergence and divergence. Analysis will be iterative, with each step of analysis informing the next.

### Data sources, data collection, and data analysis

**Cases:** Case selection will be purposive and will include laddered graduate certificate programs at a Western Canadian research-intensive university. Partners will be consulted in laddered graduate certificate programs across the university to select programs (cases) that can support the level of collaboration needed to successfully execute this research study.

**Documentation, artifacts, and archival records:** Documents, artifacts, and archival records will be collected across each case (e.g., program structure and curriculum artifacts, accreditation documentation, examples of student work, industry partner engagement documentation, meeting minutes, faculty and program evaluations) related to how laddered graduate programs are structured (inputs), implemented and experienced (process), as well as any outputs.

**Surveys:** Surveys will be developed for students, educators, alumni, industry partners, and program leaders that will be administered electronically using Qualtrics® to better understand their experiences and perceptions of engagement with graduate certificate programs. Informed consent will be assumed if participants complete and submit the survey. Surveys will be administered to,

- each cohort of students at the beginning and end of their program via their university email
- educators two times during the study via faculty contact lists
- alumni two times during the study via alumni contact lists
- program leaders one time identified by organizational leadership charts
- industry partners one time identified by program leaders.

At the end of each survey participants will be invited to participate in an interview and given the opportunity to provide their contact information for further follow-up. Quantitative survey responses will be analyzed using SPSS® (version 28) statistical software package. Changes in data over time and relationships between program structure and participant responses will be analyzed (e.g., student engagement and satisfaction) using inferential statistics [27]. Case program data will be compared to institutional data to assess for differences in metrics, such as student satisfaction and attrition.

**Interviews:** All survey participants will be invited to participate in a virtual interview via Zoom®. Semi-structured interview guides will be informed by our previous study, the survey results, and previously published literature. Purposive sampling will be used to provide for maximum variation and adequate representation in all cases. We will employ equity, diversity, and inclusivity principles when choosing interview participants to ensure appropriate representation within socio-demographic groups (e.g., age, gender, disciplines, ethnicity). Interview recruitment will continue until thematic sufficiency is reached, defined as the point at which no substantively new themes emerge across two consecutive interviews within groups. Informed consent will be obtained from each interview participant prior to the start of the interview. Interviews will be audio-recorded and transcribed verbatim. Transcripts will be uploaded to NVivo 14® for qualitative data organization. We will use inductive thematic analysis to identify common themes [28,29]. Individual researcher memos and collaborative analysis meeting minutes will be recorded as an audit trail.

**Focus groups:** All participants from across cases will be invited to participate in focus groups via Zoom® during the last year of the five-year study, following completion of individual case analysis. Focus groups will include participants from different cases; however, they will only include one type of participant (e.g., student, educator, alumni, industry partner, or program leader.) This structure is intended to reduce potential power imbalances and create a setting in which participants feel comfortable exploring their perceptions more openly. Semi-structured guides will be informed by individual case analyses. Purposive sampling will be used to ensure representation of all cases. All participants will provide informed consent. Focus groups will be audio recorded, transcribed verbatim and uploaded to NVivo 14® for qualitative data organization. We will use inductive thematic analysis to identify common themes [28,29]. Individual researcher memos and collaborative analysis meeting minutes will be recorded as part of an audit trail.

### Integration and framework development

**Fig 1:**
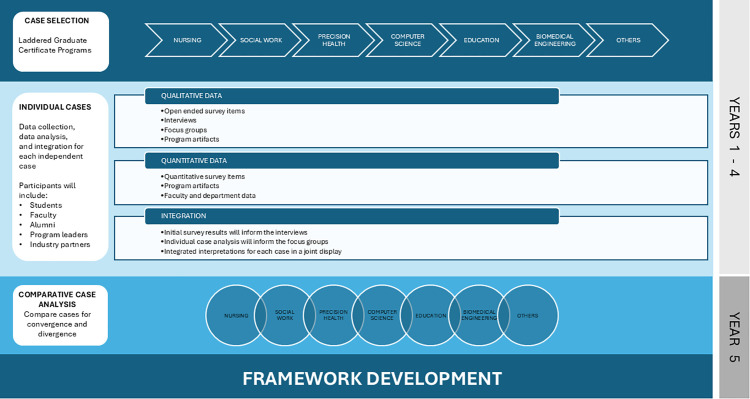
Comparative case study mixed-methods approach.

In this complex mixed methods case study, data integration will occur iteratively at multiple time points [25]. For each case, we will employ explanatory sequential sub-studies for each cohort of students and educators, alumni, industry partners, and program leader participants where survey data will inform interview recruitment and questions. In each sub-study, following individual analysis of quantitative and qualitative data, integration will occur using joint displays (Guetterman et al., 2015). Interpretations from integrated data will be used to develop individual case analysis and focus group interview guides. The cumulative findings from individual case analyses will be used to inform the comparative case analysis. Focus groups will be used to gain further insights into convergence and divergence of findings between different cases for comparative analysis. Integration will be essential as individual case studies are analyzed and will reach its pinnacle as the robust comparative case analysis takes shape by incorporating multiple data forms, from multiple stakeholder sources, over the course of four years. We will use several joint displays as analysis tools throughout this multi-year mixed methods case study to illuminate data triangulation [30] and examine common threads in the inputs, processes, and outputs that can serve as quality and innovation criteria in an overall framework. Contextual influences will be considered and included. Results will be integrated into a visual framework illustrating quality criteria for innovative laddered graduate programs development and ongoing refinement. Fig 1 offers a visual representation of the overall study.

### Expected outcomes

In rapidly changing industries, there is a need for employees to upskill to meet industry needs. Programs that are student and industry-centred have the potential to better prepare current and future industry workers to deliver innovative products and services that are beneficial to their clients. The critical knowledge gained from this research can serve as a foundation for education leaders to build new programs or revise existing programs based on factors that promote quality and innovation. Study findings are expected to benefit several target audiences, including industry partners, employees and employers, educators, students, researchers, institutional decision makers, and policymakers at provincial, national, and international levels. The findings of this research may support education leaders to build new or revise existing laddered graduate certificate programs that promote quality and innovation in teaching and learning practices and industry-focused student projects.

### Knowledge mobilization

**Fig 2:**
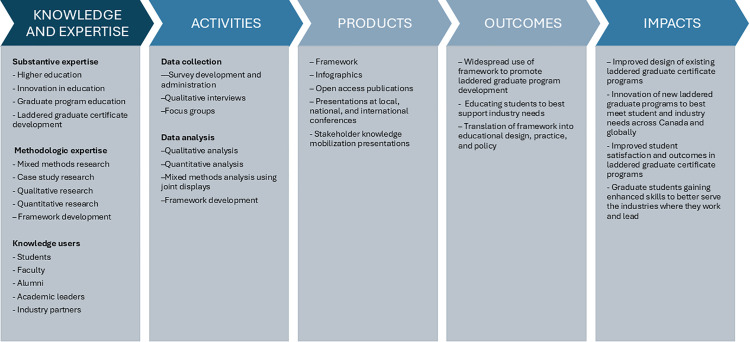
Study logic model.

Throughout this project, knowledge mobilization will be integrated as an opportunity for engagement, knowledge exchange, and co-creation of knowledge. Partners will be engaged in survey and interview guide development. Throughout the research process, collaborators from each case will be engaged to ensure this research yields meaningful and authentic findings. This collaborative approach will allow for diverse perspectives in data collection and analysis, as well as enhance knowledge mobilization. The findings of this project will be disseminated locally, nationally, and internationally to policymakers, educators, students, and industry partners through several publications in open-access journals to reach broader audiences. Fig 2 provides a logic model of the resources, activities, outputs, and intended impacts of this research study.

### Summary and significance

This research is designed to generate an evidence-informed framework that articulates the conditions to optimise student learning and program innovation within laddered graduate certificate programs. This multi-year study will be impactful not only to the students and educators, but also the clients of industry services. This research team’s collective expertise and knowledge will ensure rigorous and timely implementation of the proposed five-year research project. This will put valuable resources at the fingertips of educational leaders seeking to start innovative, student-centred laddered graduate certificate programs.
